# Correction: Two-year efficacy of varenicline tartrate and counselling for inpatient smoking cessation (STOP study): A randomized controlled clinical trial

**DOI:** 10.1371/journal.pone.0262188

**Published:** 2021-12-30

**Authors:** Kristin V. Carson-Chahhoud, Brian J. Smith, Matthew J. Peters, Malcolm P. Brinn, Faisal Ameer, Kuljit Singh, Robert Fitridge, John Litt, David Edwards, Simon A. Koblar, Jim Jannes, Antony J. Veale, Sharon Goldsworthy, Khin Hnin, Adrian J. Esterman

John Litt is not included in the author byline. John Litt should be listed as the eighth author and is affiliated with the Discipline of General Practice, Flinders University, Adelaide, South Australia, Australia. The contributions of this author are as follows: Conceptualization, Investigation, Methodology, Validation.

David Edwards is not included in the author byline. David Edwards should be listed as the ninth author and is affiliated with SA Quitline, Cancer Council SA, Adelaide, South Australia, Australia. The contributions of this author are as follows: Conceptualization, Data curation, Investigation, Resources, Validation.

The following information is missing from the Acknowledgments section: The authors would like to acknowledge the SA Quitline (and Cancer Council SA, as the provider of the Quitline) as the organisation that provided (and employed) the Counsellors who offered the pro-active call-back smoking cessation program through telephone advice.
