# Supplementary material for: Two-year efficacy of varenicline tartrate and counselling for inpatient smoking cessation (STOP study): A randomized controlled clinical trial
Source: PLoS One. 2020 Apr 29;15(4):e0231095. doi: 10.1371/journal.pone.0231095 (PMC7190140; doi:10.1371/journal.pone.0231095)
Supplement: Attachment — Submitted filename: Response to Reviewers.docx [file pone.0231095.s001.docx]

**PONE-D-19-25086 – Response to reviewer feedback**
Two year efficacy of varenicline tartrate and counselling for inpatient smoking cessation (STOP study): A Randomized Controlled Clinical Trial
PLOS ONE

Dear Editors and Peer Reviewers,

Thank you for the constructive feedback and opportunity to improve our manuscript. We have addressed each of the points in a table below.

Kind regards,

Kristin

**Journal requirements:**

| 1. Please ensure that your manuscript meets PLOS ONE's style requirements, including those for file naming. | The manuscript has been reformatted to meet the PLOS ONE’s style requirements, including those for file naming. |
| --- | --- |
| 2.  We noticed you have some minor occurrence(s) of overlapping text with the following previous publication(s), which needs to be addressed:  In your revision ensure you cite all your sources (including your own works), and quote or rephrase any duplicated text outside the Methods section. Further consideration is dependent on these concerns being addressed. | We put the manuscript through the plagiarism detection software (Turnitin), identifying some overlap with our previous 12-month efficacy publication and our published abstracts submitted to scientific conferences. Where appropriate, we have referenced the 12-month efficacy manuscript and rephrased duplicated text outside of the methods section.  In particular, we have revised the introduction opening paragraph to reduce overlap with our previous 12-month efficacy publication, added the reference to our publication for methodology, and referenced table 1 for baseline demographics.  For table 2 and table 3, we have not referenced this, as there is overlapping text related to some of the 12-week/baseline variables (not all of them as we are also presenting new variables) with the comparisons at 2-year follow-up all being completely new to this manuscript.  We have not referenced our own abstracts, which is where much of the duplication outside of our safety (Carson 2014 et.al.) and 12-month efficacy (Smith 2012) references are coming from. |
| 3. Thank you for stating the following in the Acknowledgments Section of your manuscript:  'The Department of Respiratory Medicine, The Queen Elizabeth Hospital, Adelaide, Australia'  We note that you have provided funding information that is not currently declared in your Funding Statement. However, funding information should not appear in the Acknowledgments section or other areas of your manuscript. We will only publish funding information present in the Funding Statement section of the online submission form.  Please remove any funding-related text from the manuscript and let us know how you would like to update your Funding Statement. Currently, your Funding Statement reads as follows:  'The authors received no specific funding for this work.' | We have removed funding from the manuscript. The statement of ‘no specific funding’ would be correct for the publication, as we never received any specific funding, rather the department of respiratory medicine supported the research through personnel time as part of usual practice. |

**Reviewer 1:**

| typing errors to correct, including in the summary | Amended |
| --- | --- |
| My main criticism is that varenicline placebo has not been placed in the arm with only the advice to help with weaning by phone | We have previously mentioned the lack of placebo as a limitation of this study design, and briefly touched on it again in the limitations on page 13. To reiterate this point, we have more information about the lack of placebo in the limitations section, highlighting the reason for this specific design is to provide a real-world study for use by policy makers and clinicians. |
| The second reservation, identified by the authors, is that weaning is mainly declarative. The expired CO measurement could have been done at each stage of the study and in all participants. This independent study of the laboratory that markets Varenicline shows the benefit of this molecule in long-term withdrawal and its side effects to a lesser extent than in previous studies. | Agree, this is an important limitation of this study, which has been highlighted in the discussion under limitation. We have not made any further changes at this point, however, happy to amend further if required. |

**Reviewer 2:**

| Initial inclusion: a large number of patients were excluded or not included at the beginning of the study. It would be interesting to know the main causes of non-inclusion, especially for neuropsychic diseases. This element is important since these side effects under varenicline were the subject of a debate. The good tolerance in this area under varenicline in the study may be related in part to a strict selection of patients with neuropsychological antecedents. | Unfortunately, we were not given ethics approval to capture that level of detailed information from participants. |
| --- | --- |
| Another limiting factor of the study concerns the absence of placebo versus varenicline (non-blind study) which may be negatively perceived by patients included in the group without varenicline and for health professionals. This limit is for efficacy compared to 1 and 2 years, but less alters tolerance and mortality data at 2 years (which is one of the important data in this publication). | We have previously mentioned the lack of placebo as a limitation of this study design, and briefly touched on it again in the limitations on page 13. To reiterate this point, we have more information about the lack of placebo in the limitations section, highlighting the reason for this specific design is to provide a real-world study for use by policy makers and clinicians. |
| Was there specific monitoring of varenicline patients during the first 12 weeks of follow-up? | Yes, monitoring occurred at day 3, day 5, week 1, 2, 3, 4 and 12. This has been reported in the 12-month efficacy manuscript by Smith et.al. 2012. |
| A number of patients in the varenicline-free group eventually received varenicline and / or nicotine replacement therapy, probably during follow-up. Was this element contraindicated a priori in the context of the study? | Yes, it was contraindicated. Participants were asked to only use the treatment assigned to them through randomisation. However, deviations from the protocol did occur and were monitored. This has been reported in the safety manuscript by Carson et.al. 2014. |
| Was treatment with varenicline systematically interrupted at 12 weeks or were some patients able to continue treatment for longer? | For this study, all participants reported discontinuation of treatment after 12-weeks. |
| Deaths reported in table 2 to 12 weeks appear numerically larger under varenicline (5 versus 2). Is this element significant? | No, it is not significant. ‘not statistically significant’ has been added to the text of the manuscript on page 10. |
| How many patients did they have a CO test to validate their self-reporting? | CO testing occurred in 28 VT+C participants and 23 C-alone participants. This has been reported in the Carson et.al. 2014 publication. |
| Patients in the varenicline group paid for their medication. This treatment is reimbursed in other countries. Can this element be an additional motivation factor for motivation and therefore effectiveness in this group? | Yes, we agree the addition of payment could be a motivating factor. We have now mentioned this in the limitations with supporting references relating to the economic principle of loss aversion. |
