# Supplementary material for: Two-year efficacy of varenicline tartrate and counselling for inpatient smoking cessation (STOP study): A randomized controlled clinical trial
Source: PLoS One. 2020 Apr 29;15(4):e0231095. doi: 10.1371/journal.pone.0231095 (PMC7190140; doi:10.1371/journal.pone.0231095)
Supplement: Attachment — Submitted filename: Response to Reviewers - statistical review.docx [file pone.0231095.s002.docx]

**PONE-D-19-25086R1 – Response to statistical reviewer feedback**
Two year efficacy of varenicline tartrate and counselling for inpatient smoking cessation (STOP study): A Randomized Controlled Clinical Trial
PLOS ONE

Dear Editors and Peer Reviewers,

Thank you for the constructive feedback and opportunity to improve our manuscript following assessment by the statistical reviewer. We have addressed each of the points in separate tables below.

Kind regards,

Kristin

***Major***

**Methods:**

| 1) Was this a registered trial on clinical trials.gov if so, should be stated in methods section. | Yes, it was registered with clinicaltrial.gov; Now added to methods. |
| --- | --- |
| 2) Reference of protocol should be given, not just saying published in NEJM? | Amended protocol to be the clinicaltrial.gov protocol, as this is the protocol that was being referred to (though in retrospect very ambiguously). |
| 3) More detail required on the randomisation process, i.e was it simple randomisation, any block sizes? | Simple randomisation with permuted blocks of 20, now amended in the manuscript |

**Statistical analysis**

| 1) Sample size calculation, says clustering effect was adjusted for in the sample size, the rho needs to be reported to be able to replicate the sample size? | Rho of 0.02 as per Campbell 2000 used; Now included with reference added. |
| --- | --- |
| 2) If the sample size was based on the 52 weeks, then the primary analysis should based on 52 weeks with results at 104 also presented as part of the primary outcome analysis. Otherwise this needs to be mentioned if the hypothesis is that the treatment effect would be maintained at 2 years? | The sample size calculation was only based on 52 week follow-up. This was published separately. The sample size was not powered to evaluate efficacy at 104-week follow-up. This has been reported in the manuscript. |
| 3) Needs more information about how the dependant variable is defined. | Now provided for treatment efficacy as the dependant variable. |
| 4) Analysis population, needs to be clearly defined, i.e. ITT population? | This is already reported on page 8 as being ‘intention to treat’. |
| 5) It looks like logistic regression analysis was carried out, but no mention of this in the statistical analysis section. Also based some reported results it looks like repeated measures were taken in which case, perharps more appropriate analysis is suitable to take into account the nature of the data. Also if multiple tests were done and multiple timepoints the adjustments of the p-values need to be done for these. | There were no logistic regression or repeated measure analyses undertaken. |
| 6) More detail on what baseline adjustment were made by discipline? | Amended in text to specify adjustment was for imbalance in people assigned to VT+C arm of the study from the vascular discipline. |

**Outcome and data collection**

| 1) The authors state the primary outcome was continuous outcome between weeks 2 and week 104 defined smoking at ≤ 5 cigarettes in total during the follow-up periods – was this a one-off questionnaire or question asked during the phone call to participants? Also from looking at Figure 2, this implies primary outcome was collected repeatedly at various time-points? And starting from 4 weeks instead of the mentioned from 2 weeks, please clarify. | A one-off questionnaire at 104-weeks determined the outcome of continuous abstinence for this manuscript. Re-worded in text to be clearer.  The outcome measure of 104-week continuous abstinence was not repeated at each time point, but only at 104-weeks. |
| --- | --- |

***Minor***

**Abstract:**

| 1) Abstract in results section: “odds ratio odds ratio” repeated twice. | Amended |
| --- | --- |

**Introduction:**

| 1) At the end, it would be worthwhile mentioning/distinguishing primary and secondary objectives. | This has been amended to better reflect that 104 week follow-up was a secondary objective for the study, but evaluation of this outcome was the result presented in this manuscript. |
| --- | --- |

**Results:**

| 1) It would be good add the range for age. | Added to table 1 |
| --- | --- |
| 2) If adjustments were baseline characteristics within disciplines, then it would be helpful to present (as supplementary) a baseline table stratified by discipline to the factors adjusted for are transparent. | A break-down by disciplines are already presented at the end of table 1. At this stage we don’t feel that an additional breakdown of demographic characteristics will add anything further across each of the four disciplines, above what is presented in table 1. |

**Outcome and data collection:**

| 2) [sic.] The sentence relating to participants assigned to intervention paying the full PBS subsided costs- does this mean they had to pay for their medication, would this have influenced/biased the primary outcome, in that motivation to stop smoking because they spent money themselves? I know it is acknowledged as a limitation, were the participants asked or aware during the consent process? | Yes, participants did pay for their medication, which we do acknowledge in the limitations as a potential factor influencing their quit attempts. They were aware during the consent procedure and this has been added as a sentence to study limitations. |
| --- | --- |
